# Inflammatory myofibroblastic pseudotumour of the liver in association with gall stones - a rare case report and brief review

**DOI:** 10.1186/1746-1596-5-53

**Published:** 2010-08-18

**Authors:** Talal Al-Jabri, Pandanaboyana Sanjay, Irshad Shaikh, Alan Woodward

**Affiliations:** 1 Department of Surgery, East and North Hertforshire NHS Trust, Hertfordshire, AL7 4HQ, UK; 2 Department of Surgery, Ninewells Hospital and Medical School, Dundee, Scotland, DD1 9SY, UK; 3 Department of Surgery, Brighton and Sussex University Hospitals NHS Trust, Brighton, BN2 5BE, UK; 4 Department of Surgery, Royal Glamorgan Hospital, Llantrisant, Wales, CF72 8XR, UK

## Abstract

Inflammatory myofibroblastic pseudotumours of the liver are rare tumour-like lesions that can mimic malignant liver neoplasms. The symptoms and radiological findings of this rare tumour can pose diagnostic difficulties. We describe a 69-year-old gentleman who was admitted to our department with symptoms suggestive of acute cholecystitis. Ultrasonography and computed tomography of the liver raised the possibility of metastatic liver disease. A core biopsy of the liver was performed to confirm the diagnosis of liver metastasis. Unexpectedly it showed no evidence of malignancy but instead revealed an inflammatory myofibroblastic pseudotumour of the liver. This case report highlights the diagnostic dilemma that arose due to the similarity of appearances between the two pathological entities on imaging and this stresses the need for accurate histological diagnosis so as to avoid unnecessary surgical intervention. To the best of our knowledge, only a minority of cases are reported in the literature associating a hepatic inflammatory myofibroblastic pseudotumour with gall stones.

## Background

An inflammatory myofibroblastic pseudotumour (IMFP) of the liver (also known as a plasma cell granuloma) is a rare, benign, tumour-like lesion first described in 1953 by Pack and Baker [1]. Histologically, it is characterized by the presence of proliferating fibrovascular tissue mixed in with a heterogenous population of acute and chronic inflammatory cells mainly consisting of plasma cells, lymphocytes and occasionally histiocytes [2]. The symptoms and radiological findings of this rare tumour can mimic malignancy and pose diagnostic difficulties. IMFPs most often occur in the lung, however have also been reported in the central nervous system, orbit, and liver [3]. We describe an unusual case of a 69 year old man known to suffer with rheumatoid arthritis, who presented with symptoms of cholecystitis.

## Case Presentation

A 69-year-old Caucasian gentleman presented to the surgical department with right upper quadrant pain, nausea, vomiting and a recent weight loss. His medical antecedents included rheumatoid arthritis and bronchiectasis. On examination, the patient was found to have tenderness in the right hypochondrium. An abdominal ultrasound (US) scan revealed a thick-walled gall bladder with multiple calculi and a normal looking liver. Laboratory investigations showed a decreased haemoglobin concentration (11.6 g/dl), an elevated C-reactive protein level (137 mg/l) and an increased erythrocyte sedimentation rate (98 mm/hr). The liver function tests were deranged with a raised alkaline phosphatase (412 u/l), a raised alanine transaminase (53 u/l) and a normal serum bilirubin level. An initial diagnosis of cholecystitis was made and the patient was treated empirically with antibiotics.

**Figure 1:**
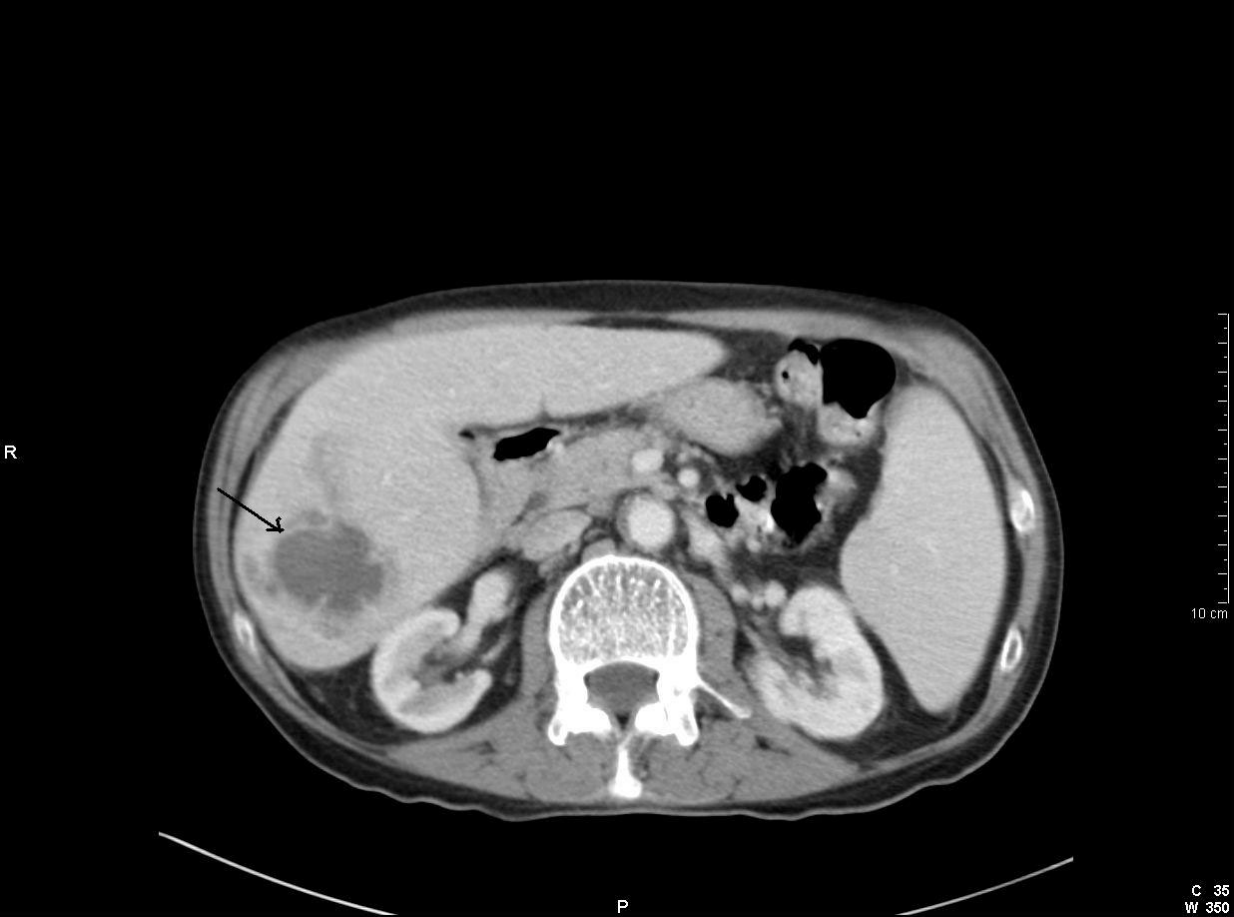
**CT scan demonstrating several ill-defined low attenuation lesions with some peripheral enhancement**.

**Figure 2:**
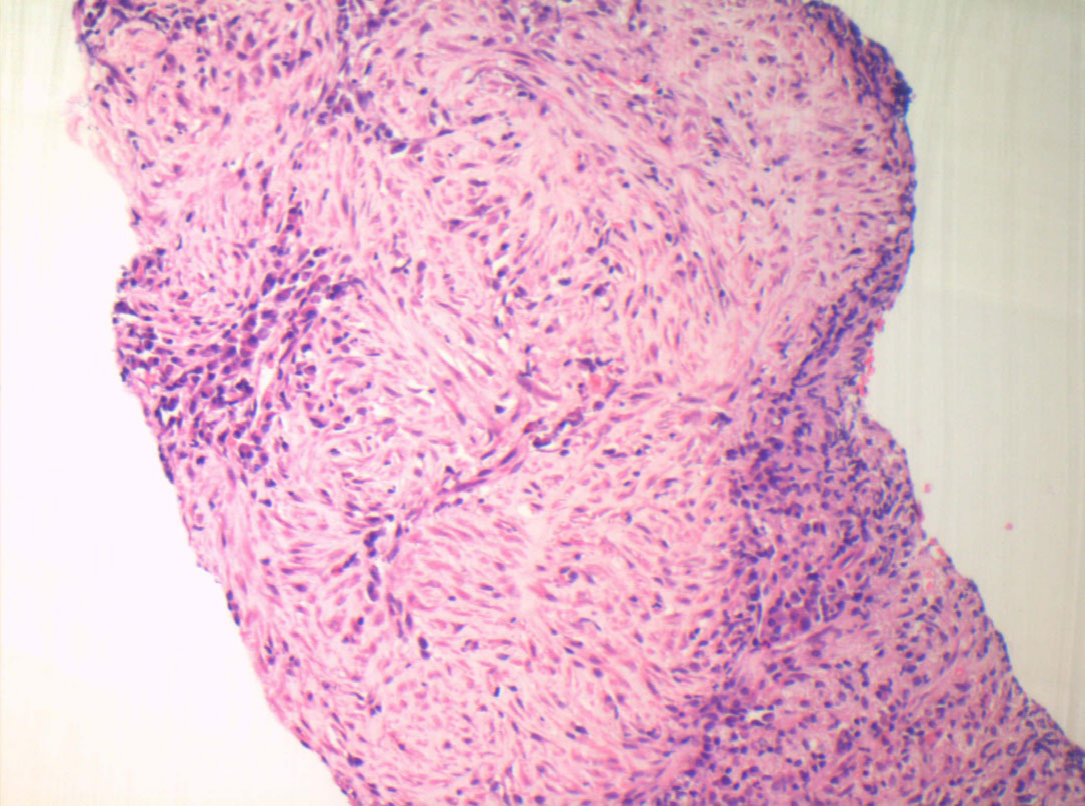
**Medium power histology) showing a bland spindle cell lesion with multiple foci of chronic inflammatory cells (predominantly mature plasma cells) (X 20, Magnification, H&E stain)**.

**Figure 3:**
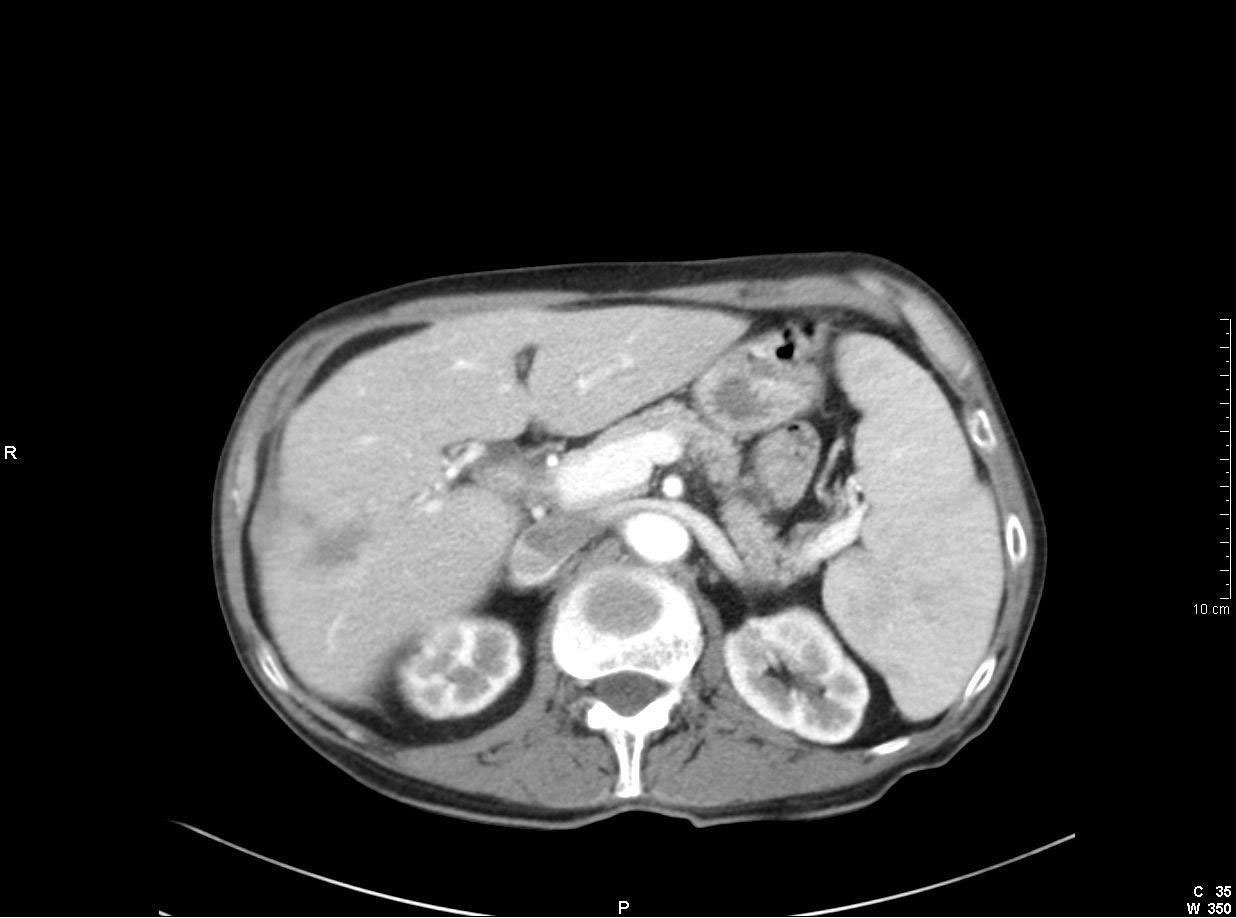
**CT scan showing a marked reduction in size of the hepatic lesions**. A small organised subcapsular haematoma is noted as result of previous biopsy.

Whilst waiting for a laparoscopic cholecystectomy, the patient once more presented to the emergency department with recurrent episodes of abdominal pain. A repeat abdominal US scan revealed an ill-defined area in the right lobe of the liver with altered echogenicity. This ambiguous region was considered to represent either metastatic infiltration or an early hepatic abscess. Subsequently, computed tomography (CT) revealed multiple low attenuation lesions in the right lobe of the liver, the largest measuring 5.5 cms (Fig 1). The lesions demonstrated patchy central and peripheral enhancement with contrast. A provisional diagnosis of liver metastases from an unknown primary was made. However, US guided fine needle aspiration (FNA) of the liver demonstrated the presence of benign hepatocytes, acellular debris and a mixture of acute and chronic inflammatory cells. Surprisingly, no malignant cells were identified. A core biopsy of the liver revealed predominantly bland spindle-celled stromal tissue with cellular infiltrate rich in plasma cells (Fig 2). There was no evidence of malignancy in the tissue examined. A final diagnosis of an IMFP of the liver was made. During the course of the investigations the patient had complete symptomatic relief on oral antibiotics. The patient was followed up with a repeat CT scan 3 months later which showed a marked improvement with an almost complete resolution of the lesions (Fig 3). The patient is asymptomatic and remains well.

## Discussion

An IMFP is a benign, tumour-like mass characterized by proliferating fibrous tissue infiltrated by inflammatory cells [2]. This condition is known to occur in several organs including lymph nodes, spleen, brain, spinal cord, larynx, thyroid gland, breast, pancreas, gastrointestinal (GI) tract and bladder, but most frequently it occurs in the lungs and liver [3]. Hepatic IMFPs occur predominantly in the right lobe of the liver although multicentricity has been described [4] and it has previously occurred in the gall bladder fossa of the liver [5].

The etiology of IMFPs remains unclear although infection, radiation and chemotherapy have been suggested as possible causes [6] . More recently it has been associated with chronic inflammatory and autoimmune disorders [3,4], stem cell transplantation[5], Crohn's disease [5], gastro-intestinal stromal tumours [7], congenital neutropenia [8] and pregnancy [9]. This condition can present as an abdominal mass or with non specific symptoms such as intermittent fever, abdominal discomfort, weight loss and malaise. The other presentations include obstructive jaundice [10], splenomegaly, and portal hypertension [11]. Laboratory investigations often indicate an ongoing inflammatory process with a leukocytosis and a raised ESR and CRP, as was evident in our case.

IMFPs most often occur in childhood and early adulthood in non-European populations [12]. The male to female ratio has been previously reported between 1:1 and 3.5:1 [10,12]. Distinctively, our patient was 69-years-old and of European descent.

Radiological diagnosis of this tumor can be demanding, sonographically the lesion may be hyperechoic, hypoechoic, or complex in echogenicity. CT imaging may help alleviate the diagnostic dilemma common to patients presenting with IMFPs. Enhancement patterns between hepatocellular carcinoma (HCC) and IMFPs differ significantly, with HCCs exhibiting hyperdensity in the arterial phase and hypodensity of the capsule in the delayed phase. On MRI the lesion demonstrates increased signal intensity on both T-1 weighted and T-2 weighted sequences in relation to a normal liver [13]. These variable appearances on imaging stress the need for accurate diagnosis of the tumor by biopsy. The differential diagnoses on imaging include liver abscesses, metastasis, peripheral cholangiocarcinoma and hepatocellular carcinoma. In order to confirm the diagnosis further, a histological diagnosis of an IMFP can be made either by FNA or by core biopsy. It is important to note that despite FNA, IMFPS have been misdiagnosed as malignant tumours in the past. This is most likely due to the difficulty in assessing fibrous lesions with FNA [14].

Histologically, IMFPs tend to appear as densely hyalinized structures with whorl-like patterns of dense collagen bundles infiltrated by multiple inflammatory cells. No features of malignancy are present. There is great histological variation and the features of IMFPs tend to be non-specific. This has led to IMFPs becoming a diagnosis of exclusion in adults [14].

To the best of our knowledge, only a minority of cases associating hepatic IMFP with gall stones are reported in the literature [12]. The exact etiological link between the two is uncertain. However, the presence of gall stones could potentially enhance entry of enteric bacteria into the portal circulation, which might then result in an inflammatory response and possibly an IMFP.

The management of an IMFP of the liver has traditionally been surgical but more recently spontaneous regression of the tumour has been reported with the use of antibiotics or non-steroidal anti-inflammatories as was evident in our patient [11,12,15]. Surgery is recommended to relieve obstructive jaundice due to IMFPs. Currently, there are no clinical studies comparing the outcomes of patients treated conservatively and those treated with surgery. Also, there is no data regarding the duration of follow up in patients managed conservatively [16]. More recently local recurrence and distant metastases have been attributed to the ploidy of the lesion with aneuploid lesions more likely to recur and metastasize than diploid lesions [17]. Therefore flow cytometric DNA analysis of these tumours has been suggested to provide the clinician with both diagnostic and prognostic information and to individualize the most feasible therapeutic approach [18].

In conclusion this case report emphasizes the importance of considering an IMFP as part of the differential diagnosis and highlights that a conservative approach to a patient with an IMFP is an effective treatment option.

## Abbreviations

IMFP: Inflammatory Myofibroblastic Pseudotumour; US: Ultrasound; CT: Computed Tomography; ESR: Erythrocyte Sedimentation Rate; CRP: C - reactive protein; GI tract: Gastrointestinal tract; FNA: Fine Needle Aspiration: HCC; hepatocellular carcinoma.

## Competing interests

The authors declare that they have no competing interests.

## Authors' contributions

PS, IS, AW and TAJ were involved in the acquisition of data and it's analysis. PS, TAJ and IS wrote and edited the manuscript. All authors have read and approved the final manuscript.

## Consent

Written informed consent was obtained for the publication of this article and the accompanying images. A copy of the written consent is available for review by the Editor-in-Chief of this journal.
